# Healthy ageing begins with a healthy lifestyle

**DOI:** 10.1016/j.ebiom.2023.104528

**Published:** 2023-03-10

**Authors:** eBioMedicine

Throughout the ages, humans have searched for means and magic elixirs to live longer. Nowadays, the principles of healthy ageing are of global interest and relevance, and are supported by biomedical research. According to WHO estimates, the number of people older than 60 years in 2050 will be 2.1 billion, 1.7 billion of whom will be living in low-income countries. In response to the rapidly increasing population of people older than 60 years, the UN General Assembly declared 2021–2030 the UN Decade of Healthy Ageing to promote longer and healthier lives. As population ageing is becoming a global issue, finding accessible and affordable interventions to promote healthy ageing will be crucial. Healthy lifestyle choices will probably be essential.

Ageing is associated with the onset of various chronic diseases, such as cardiovascular diseases and dementia. Caloric restriction has been shown to reduce incidence of age-related chronic disease rates and increase lifespan in animal studies. In the September, 2019, issue of *The Lancet Diabetes & Endocrinology*, William Kraus and colleagues reported the first human study of medium-term caloric restriction. They assigned young and middle-aged (aged 21–50 years), healthy, non-obese male and female participants to a 25% caloric-restriction diet group (n = 143) or an ad libitum control group (n = 75) and followed up for 2 years. Caloric restriction achieved a mean reduction in calorie intake of 11.9%. Compared with the participants in the control group, those in the caloric-restriction diet group had persistent and significant reduction of all measured conventional cardiometabolic risk factors, including LDL-cholesterol, total cholesterol to HDL-cholesterol ratio, and systolic and diastolic blood pressure. Furthermore, no serious adverse events were reported, suggesting that moderate caloric restriction can be a safe and effective intervention to promote cardiometabolic health. In the February, 2022, issue of *Science*, Olga Spadaro and colleagues further analysed the data from Kraus and colleagues to investigate the effects of caloric restriction on immunometabolism. 2 years of caloric restriction promoted thymopoiesis and induced pathways regulating mitochondrial bioenergetics, anti-inflammatory responses, and longevity. These findings show the mechanisms by which caloric restriction regulates lifespan and could help develop precise interventions for improving healthy ageing if a caloric-restriction diet is not suitable.

Fluid intake could also contribute to the ageing process. Natalia Dmitrieva and colleagues reported in the January, 2023, issue of *eBioMedicine* that middle-aged individuals (aged 45–66 years) with high normal serum sodium levels (142 to <146 mmol/l) had a 39% increased risk of developing chronic diseases, including heart failure, dementia, chronic lung diseases, and stroke. The biological age of the participants with this range of serum sodium levels was up to 50% more likely to be older than their chronological age, which was associated with an increased risk of chronic diseases and premature mortality. As serum sodium concentration has been used as a proxy for hydration status in the healthy human body, this study suggested that a proper increase of fluid intake to maintain optimal hydration might slow the ageing process.

Caloric restriction aims to control energy intake to the body, but another option to maintain energy homoeostasis would be to increase energy expenditure (eg, to increase physical activity or exercise). As different types of physical activity require different skills and capacities, one more suitable for people older than 60 years to reduce mortality should be established. In the August, 2022, issue of *JAMA Network Open*, Eleanor Watts and colleagues studied the associations of seven types of physical activities (ie, running, cycling, swimming, racquet sports, golf, walking for exercise, and other aerobic exercise) with all-cause, cardiovascular, and cancer mortality in more than 270,000 participants aged 59–82 years. Each activity showed a curvilinear dose–response association with mortality risk. Low metabolic equivalent of task (MET) hours per week (0.1 to <7.5 or 7.5 to <15) of any activity type was associated with a large reduction in mortality risk, with diminishing returns for each increment in activity thereafter. Compared with those who did not participate in any activity, 7.5 to less than 15 MET h per week of racquet sports (HR 0.84, 95% Cl, 0.5–0.93) and running (0.85, 0.78–0.92) were associated with the lowest risk for all-cause mortality. For cardiovascular mortality, racquet sports (0.73, 0.59–0.89) was associated with the greatest risk reduction and running (0.81, 0.69–0.95) was the most effective activity to reduce cancer mortality. Although excessive physical activity might bring more harms than benefits for people older than 60 years, this study suggested that 7.5 to less than 15 MET h per week of any activity is sufficient to reduce mortality risks, with racquet sports and running being the most beneficial.

Social factors can also influence aspects of the ageing process, such as cognition. In the November, 2022, issue of *The Lancet Healthy Longevity*, Suraj Samtani and colleagues reported the largest meta-analysis to date of the association between social connections and cognition at the individual participant level, which included 13 longitudinal cohort studies globally and more than 38,000 participants. Living with other people predicted slower global cognitive, memory, and language decline than living alone, and weekly interactions with family and friends and weekly community group engagement predicted slower memory decline than no interactions and no engagement. These findings emphasise the importance of social connections for the mental health of people older than 60 years, and might encourage family members and the community to offer more group activities for older members.

A healthy lifestyle is an integration of different healthy behaviours. In the January, 2023, issue of *BMJ*, Jianping Jia and colleagues reported the effect of an optimal lifestyle on memory loss, one of the phenotypes in ageing, in a prospective cohort of more than 29,000 people older than 60 years. They assessed six healthy lifestyle factors, including a healthy diet, regular physical exercise, active social contact, active cognitive activity, never or previously smoked, and never drinking alcohol. Participants were categorised into three groups on the basis of how many factors were present: the favourable group (4–6 factors), the average group (2–3 factors), and the unfavourable group (0–1 factor). Regardless of apolipoprotein E ε4 status, participants in the favourable and average groups had slower memory decline than those in the unfavourable group. This study highlights the importance of a heathy lifestyle in neurocognitive health. Whether it can affect other processes in ageing requires further investigation.

Ageing is a complex process, much of which remains elusive. The aims of healthy ageing are not only to extend lifespan, but to improve physical and mental health and quality of life of people older than 60 years. A healthy lifestyle is not the only method to promote healthy ageing, but can be an easy way. With the rapid growth of the population of people older than 60 years, understanding the mechanisms of ageing and developing interventions to prevent or delay the onset of age associated diseases are becoming increasingly important and urgent. *eBioMedicine*, as a translational publishing platform, welcomes all basic and clinical research on healthy ageing.

